# Measuring Client Experiences in Maternity Care under Change: Development of a Questionnaire Based on the WHO Responsiveness Model

**DOI:** 10.1371/journal.pone.0117031

**Published:** 2015-02-11

**Authors:** Marisja Scheerhagen, Henk F. van Stel, Erwin Birnie, Arie Franx, Gouke J. Bonsel

**Affiliations:** 1 Erasmus Medical Centre, Department of Obstetrics and Gynaecology, Division of Obstetrics and Prenatal Medicine, Rotterdam, The Netherlands; 2 University Medical Center Utrecht, Julius Center for Health Sciences and Primary Care, Department of Health Technology Assessment, Utrecht, The Netherlands; 3 Institute of Health Policy and Management, Erasmus University Rotterdam, Rotterdam, The Netherlands; 4 University Medical Center Utrecht, Division Women and Baby, Department of Obstetrics, Utrecht, The Netherlands; Kuopio University Hospital, FINLAND

## Abstract

**Background:**

Maternity care is an integrated care process, which consists of different services, involves different professionals and covers different time windows. To measure performance of maternity care based on clients' experiences, we developed and validated a questionnaire.

**Methods and Findings:**

We used the 8-domain WHO Responsiveness model, and previous materials to develop a self-report questionnaire. A dual study design was used for development and validation. Content validity of the ReproQ-version-0 was determined through structured interviews with 11 pregnant women (≥28 weeks), 10 women who recently had given birth (≤12 weeks), and 19 maternity care professionals. Structured interviews established the domain relevance to the women; all items were separately commented on. All Responsiveness domains were judged relevant, with Dignity and Communication ranking highest. Main missing topic was the assigned expertise of the health professional. After first adaptation, construct validity of the ReproQ-version-1 was determined through a web-based survey. Respondents were approached by maternity care organizations with different levels of integration of services of midwives and obstetricians. We sent questionnaires to 605 third trimester pregnant women (response 65%), and 810 women 6 weeks after delivery (response 55%). Construct validity was based on: response patterns; exploratory factor analysis; association of the overall score with a Visual Analogue Scale (VAS), known group comparisons. Median overall ReproQ score was 3.70 (range 1–4) showing good responsiveness. The exploratory factor analysis supported the assumed domain structure and suggested several adaptations. Correlation of the VAS rating and overall ReproQ score (antepartum, postpartum) supported validity (r = 0.56; 0.59, p<0.001 Spearman's correlation coefficient). Pre-stated group comparisons confirmed the expected difference following a good vs. adverse birth outcome. Fully integrated organizations performed slightly better (median = 3.78) than less integrated organizations (median = 3.63; p<0.001). Participation rate of women with a low educational level and/or a non-western origin was low.

**Conclusions:**

The ReproQ appears suitable for assessing quality of maternity care from the clients' perspective. Recruitment of disadvantaged groups requires additional non-digital approaches.

## Introduction

Performance of maternity care is primarily determined by its health outcomes, in particular mortality and morbidity of mother and child over the short and long term. Such outcomes differ globally, countrywise, and also within countries where health care quality differences may be in part responsible [1–5].

Another dimension of maternity care performance, is the way that clients (here primarily the women involved) experience the care provided. This includes whether they feel secure, feel treated with respect, feel adequately informed; are facilities in a broad sense accessible and client-friendly. These client experiences with health care provision are supposed to be important for two reasons: 1) client experiences represent an independent outcome of performance, which may guide choices of health care provider if outcomes are similar [6]; 2) client experiences may affect clinical outcomes through several ways, hence may act as determinant of the aforementioned outcomes in mother and child [7–10]. According to the World Health Organization (WHO), which developed an influential concept to measure client experiences, adequate client orientation ultimately relates to respecting human rights, specified for the context of health care provision [6,11,12].

To achieve uniform measurement of client experiences as a performance indicator, the WHO elaborated the so-called Responsiveness model, after comprehensive preparatory studies and consultation. Following this model, responsiveness is defined as the way a client is treated by the professional and the environment in which the client is treated, where eight different domains are suggested to cover the concept. This model deliberately focusses on individual experiences rather than characteristics of processes or structures, acknowledging that between and even within countries the same client experiences may be arrived at by various means. The model has been shown to enable comparison of experienced performance within and between countries on a general level [6,13].

So far, the responsiveness questionnaires were never specified to a health care subsystem, such as maternity care. We selected the WHO responsiveness model to measure client experiences in maternity care in the Netherlands, for reasons explained below.

Measurement of maternity care performance in general is a challenge, because maternity care consists of different services (e.g. antenatal check-ups, care during the delivery); different time windows (antepartum phase, childbirth, postpartum phase) and involves several professions; and professionals (e.g., obstetricians, midwives, and maternity nurses) where many tasks are executed interchangeably.

Seen from the client's perspective, the health system in many countries shows considerable variety in health care arrangements, the location of organizations (e.g. urban vs. rural), and overall integration.

This is particularly true in the Netherlands where currently the maternity care is changing from a two-tier system to an integrated care system [14–18]. The current dominant two-tier system is based on strict division of tasks, with primary care though midwives and general practitioners for assumed low-risk pregnant women, and secondary/tertiary care for assumed high-risk women in hospitals and perinatal centers. Primary care and secondary care professionals each have their own professional autonomy, responsibilities, and financial arrangements, and integration of processes and risk standards is limited. In view of the unsatisfactory performance of the Dutch maternity care system (perinatal outcome, maternal outcome, system weaknesses e.g. in risk management and 24/7 hospital quality), maternity care shifts towards integrated care, following the 2010 advice of a National Committee on Perinatal Care established by the Ministry of Health [1,2,18–20]. Integrated care combines the delivery and organization of health services; it assumes one clinical perspective, one risk management approach and one client orientation [21].

Existing indicators and questionnaires all appeared limited for our purposes. They either focus on specific processes, monodisciplinary perspectives or assume a specific maternity care organization; they usually contain additional modules on outcomes and procedural facts, and lack a formal aggregate scoring system for the client's experience allowing a graded quality judgment[22]. For example, the questionnaires of the British National Health Service (Women's Experience of Maternity Care) [22] and the National Perinatal Epidemiology Unit [23] include only part of the responsiveness domains, focusing on the personal quality of services. The Dutch Consumer Quality Index for primary maternity care [24] and a similar survey for postnatal care [25] focus on the care delivered by one professional group (community midwife, maternity nurse) for specific phases (antenatal, delivery, first postnatal week) assuming monodisciplinary care as standard, i.e. without any involvement of hospital, gynaecologist or paediatrician. Two other comprehensive interviewer-based instruments are obviously not suited for self-report. The Maternity Experiences Survey from Canada assumes additional explanatory support of an interviewer, and its length precludes routine application [26]. Prior to the ReproQ, we developed a structured face-to-face interview based on the WHO responsiveness concept to evaluate care in an integrated birth centre, which includes clinical postdelivery services [27]. Like the Maternity Experiences Survey this interview was too long for routine application, and results suggested that after a complicated delivery, bias could occur in the report of client experiences antenatally (“carry back” effect [28]). Other surveys, not listed here, primarily ask for the presence of structural features or care processes rather than for the performance-as-experienced. International comparisons of health services [29] have made clear that one cannot easily rely on the structural features, as a proxy for the actual client centeredness of services, in particular in case of disadvantaged groups. The WHO model seemed appropriate and suitable in this case as starting point for a uniformly applicable questionnaire on client experiences, as it allows for measurement regardless of the particular organizational and professional characteristics. We expect that this questionnaire is sensitive for performance characteristics that benefit from integration, such as–in terms of the WHO domains–communication, prompt attention, information continuity, etc. The questionnaire may also be sensitive for potential negative aspects of integration such as decreased autonomy if care becomes more rule-based. Existing indicators and questionnaires either focus on processes and structural features (from a professional point of view) of maternity care, or are to some extent restricted to one organizational structure [22–24], justifying our comprehensive approach on the base of a proven concept.

The study presented here describes the development of a client experiences questionnaire on the basis of the WHO responsiveness model, and presents basic psychometric evidence.

## Methods and Materials

**Figure 1:**
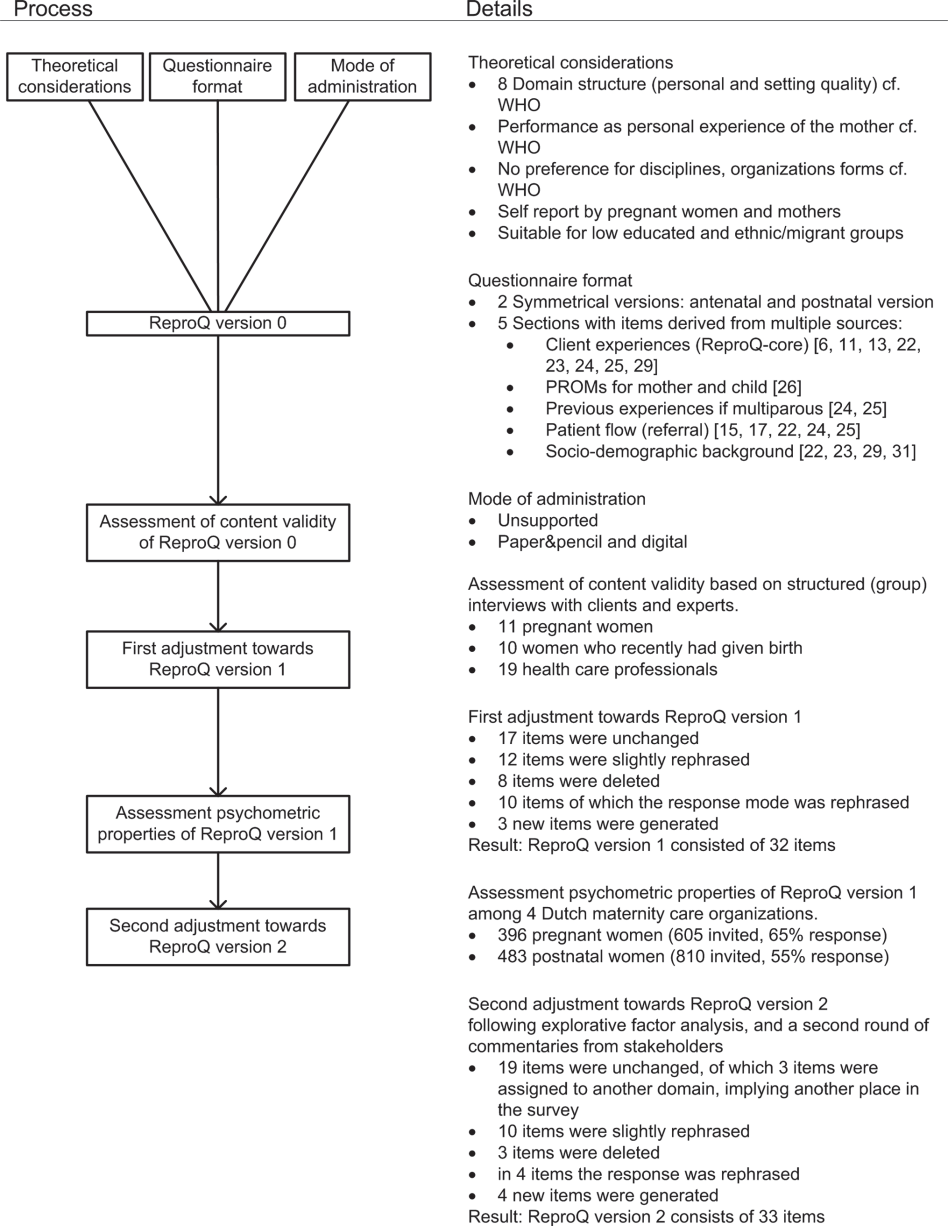
Flowchart of the developmental process of the ReproQ.

The development of the questionnaire, called the ReproQ, covered three phases: 1. overall design and specific item generation for the client experiences following the WHO concept; 2. interview study involving relevant stakeholders to determine the content validity of the null version of the ReproQ; 3. survey study in 4 different regions to enable constructive psychometric analysis. Prior to the description of the methods used in these phases, we describe the seven theoretical considerations on which the ReproQ is based. The phasing is shown in Fig. 1.

### Theoretical considerations

**Content:** 1) The WHO responsiveness model was the conceptual basis. This model consists of four domains concerning the interaction between the client and health professional (dignity, autonomy, confidentiality, and communication), and four domains concerning the organizational structure (prompt attention, access to family and community support, quality of basic amenities, and choice and continuity of care) [6,13].

2) In agreement with the WHO model, the operationalization of the concept into experience items avoided any implicit preference toward provider or organization structures, leaving room to different organization structures and different levels of integrated care (high/low). We did not measure integral working as such; moreover, we assumed performance in terms of the WHO responsiveness concept would benefit from more integration, if performed well.

3) The questionnaire focussed on performance-as-experienced by the client, rather than on structural features or processes.

**Coverage:** 4) The mother is the principle bearer of experiences, because choices and decision-making in maternity care delivery generally rest with the mother or mother-to-be. In addition, the child's father may not invariably be a desirable or available co-respondent. Obviously, responsiveness cannot be reported by the neonates themselves.

5) From a system's point of view, maternity care actually consists of service delivery that is different during pregnancy, during childbirth and postpartum care. The antepartum phase can be defined as monitoring intermittent preventive care, mostly in an ambulatory facility. Screening is a particular feature at onset of antenatal care. The delivery is a single, high impact process, which shows many features of acute curative care. Postpartum care aims at monitoring the health of both mother and child, and at empowering the parents for the future. In these three phases, the interaction with health care professionals, facilities, and the time axis of experiences are quite different. We developed two “mirror” versions of the questionnaire; one to measure experiences during pregnancy (antepartum) and one to measure experiences during delivery and thereafter (postpartum).

Both versions are symmetrical, in that the same type of experiences are asked for and the way these are asked for is also identical, yet each item is adapted to the context (antepartum vs. postpartum). In each version we asked the client to judge each item during two reference periods: in the antepartum questionnaire the first and second half of pregnancy, in the postpartum questionnaire the event of labour and birth, and the subsequent postpartum week. Consequently, responses on all responsiveness items existed for 4 different reference periods.

**Feasibility:** 6) The questionnaire was intended for self-report of clients, without support, and was primarily developed as online survey. A paper version should be also available, limiting the possible complexity of the digital version.

7) The questionnaire was suitable for clients with low educational level (defined as duration ≤6 years for migrant women and ≤8 years for women of Dutch origin) and migrants and clients of non-western origin. This was achieved by the following: a) the response mode uniformly used 4 simple categories: “never”, “sometimes”, “often”, and “always”, with a numerical range of 1 (worst) to 4 (best); b) items consist of short sentences; c) common language was used (reading level B1, checked by word frequency lists [30]); testing by members of the target group. We are aware that illiterate clients need another approach, most likely a structured interview.

### Survey structure and item generation

The questionnaire consists of five sections, i.e.: 1) information about the current care process, the location of care (e.g. home or hospital) and the dominant health professional delivering care (e.g. midwife or obstetrician); 2) the clinical outcome of both mother and child, as perceived by the mother in non-medical terms; 3) the client experiences in terms of the eight key domains of the responsiveness model; 4) information about previous pregnancies; 5) socio-demographic characteristics of the client.

Section 3 is the key section of the ReproQ. For the generation of the items of this section we used four sources. First we looked at the responsiveness items of the World Health Survey and Multicountry Survey model [6], adapting items with contextual information of maternity care. Second, we used items generated for a previously developed face-to-face interview [27]. Third, we explored published questionnaires on the same or related concepts concerning maternity care [22–24]. Finally, we used the manual of the Dutch Consumer Quality Index method to measure client experiences [31].

The other sections were developed to enable interpretation of the experiences, and supplementary discriminative content validation, as reported in this paper. The elaboration of these sections was based on existing formats and will not be discussed further.

### Content validity: interviews

Content validity of the pilot version of the ReproQ (version 0) was determined through structured interviews, supported by questionnaires, with 11 pregnant women, 10 women who recently had given birth (≤12 weeks postpartum) and 19 maternity care professionals (7 midwifes, 4 obstetricians, 2 maternity nurses, 4 executives and 2 perinatal health officers). In Spring 2012, the participating clients were approached in three different maternity care organizations in The Netherlands with different levels of integration: 1) a fully integrated midwifery practice and a peripheral hospital (Roosendaal); 2) a fully integrated midwifery practice and a university hospital (Utrecht); 3) a clinic from the university hospital in Rotterdam, with an adjacent birth centre (Rotterdam). The hospitals involved, and the birth centre provided care to clients of several associated primary care midwifery practices and clients, which were already under care from the hospital. Clients were approached either by their professional or a member of the research team. The maternity care professionals were recruited from the same facilities through their team manager.

We intended to perform a group interview with each group of relevant stakeholders in each center, resulting in altogether nine group interviews. We intended to include a minimum of six participants per interview. All interviews were chaired and performed by the research team. The number of participants for each organization is shown in Table A1 in the S1 Table.

The group interviews of about 2 hours followed a common structure: 1) prioritisation of the responsiveness domains; 2) two comments on each item (a. contents and b. grammar/readability); these were first written down for each item separately, and subsequently discussed in plenum; 3) systematic check for missing topics or perspectives of the questionnaire. Health professionals were additionally asked to rate the suitability of the experience items of the questionnaire (ReproQ core, section 3) from the perspective for women with a low educational level and non-western women. Because they regularly encounter many of these women during their consultation hours, we assumed that they could give a reasonable judgment of the suitability. They separately rated the suitability for women with a low educational level and for women with different ethnicities on a five point scale [strongly agree-strongly disagree].

More in detail, the client interview first invited the participants to individually describe their wishes and possible improvements concerning the maternity care they had received. Discussion could follow. Second, clients were asked which two of the eight Responsiveness-domains were most important to them. Finally, the clients were asked to fill out the null version of the questionnaire; comments were noted and discussed plenary. Each group client interview lasted about 2 hours. We performed some individual interviews, when the number of participating clients was less than the required six participants per group interview. Each participant received a compensation of €20 ($27, £16).

The group interviews with maternity care professionals lasted on average 1.5 hours and were unrewarded. In the group interviews with clients, 7 pregnant women and 9 women who recently had given birth participated. In addition, we interviewed 4 pregnant women and 1 woman who recently had given birth individually. In the group interviews with health professionals, 7 midwifes, 4 obstetricians, 2 maternity nurses, 4 executives and 2 perinatal health officers participated.

The null version of the ReproQ was adjusted based on the joint comments, where comments of clients and health professionals were regarded as equally relevant. We assumed that the item content to be valid if the comments involved no or minor changes in item wording or response categories.

### Survey study to obtain psychometric characteristics

We obtained psychometric characteristics of the adjusted questionnaire in a subsequent survey study. Pregnant women and women who recently had given birth were asked for participation when they visited their care provider. After written informed consent, they received an invitation by email to fill out the web-based questionnaire. Patients were locally recruited with the support of the organisation.

To qualify for the antepartum questionnaire, women should have a gestational age less than 34 weeks; to qualify for the questionnaire concerning the delivery and postpartum care, women should have given birth less than 6 weeks earlier. The antepartum questionnaire was sent in the 34^th^ week of their pregnancy, the postpartum questionnaire was sent 6 weeks after the expected date of delivery. Non-responding women received an e-mail reminder 2 weeks after they received the initial questionnaire.

Four maternity care organizations participated for client recruitment. Three of these also participated in the interview study. The additional organization included four hospitals and four midwifery practices.

Altogether a wide range of organisational structures and client populations was covered. To determine the psychometric characteristics of the questionnaire, we aimed at a minimum of 300 completed antepartum and 300 completed postpartum questionnaires. Because the questionnaire exists of two versions, that are not identical, we aimed at a sample size of 300 respondents for both versions of the questionnaire. The sample size was based on the Dutch manual to develop Consumer Quality questionnaires [31].

### Analysis

**Interviews relevant stakeholders to determine the content validity**. The prioritised domains will be reported in percentage of domains ranked first or second.

The items were primarily adapted based on the detailed individual written comments. Combining the comments per item resulted in 1) items needing no change; 2) items to be simplified or changed to avoid textual ambiguities; 3) adaptation of the response mode in specific cases, e.g. through addition of the option “not applicable”, or changes in the labels of the response levels; 4) items to be removed, if the item did not sufficiently fit to the concept or if the item showed too much overlap with other items questions

The comments on missing domains or items are reported if multiple comments indicated such missing.

The response mode of the five point suitability-questions for women with a low educational level, and of non-Dutch origin were later reduced to three categories: agree-neutral-disagree, as extreme categories were rarely used.

**Survey study followed by psychometric analyses**. We invited 605 pregnant women, of whom 396 responded (65%), and invited 810 women who recently had given birth, of whom 483 responded (55%). We excluded 45 pregnant women and 50 women who recently had given birth, because 50% of their answers were missing in 2 of more domains. The first step in the analysis was the checking for response patterns, such as a floor-ceiling-effect, the computation of the percentage missing-values per item, and the computation of the digitally measured response time. The second step involved analysis of the construct validity using Exploratory Factor Analysis (EFA) [32]. The main goal was to identify items that required replacement to another domain, rewording, or removal. Because we use a so-called formative measurement model (pre-stated domain structure) and not a reflective model, the decisions on which item belongs to which domain finally are based on content and the EFA combined, rather than EFA alone.

The analyses were intended to be performed separately for the four phases of maternity care, namely first half pregnancy, second half pregnancy, birth, and postnatal care. However, as answer patterns for the first and second half of the pregnancy were close to identical, we only present data of the second half of pregnancy, and data of birth and postnatal care (3 reference periods).

In the EFA for labour and birth, and postnatal care, the three questions of the domain Basic Amenities were not included, because the number of respondents was too small due to routing in the questionnaire. The EFA was conducted as a principal components analysis followed by orthogonal rotation (Varimax) [32]. The factors were determined by the Kaiser criterion (i.e. an Eigenvalue > 1). In addition, we computed Cronbach's alpha to determine the internal consistency of each factor. Note that internal consistency of items may be empirically low despite a close relation in terms of contents: e.g. items on the accessibility all refer to one basic concept, yet the travelling distance to the facility is not empirically associated to the accessibility by phone.

Third, convergent validity was tested by the association between an overall 10-point VAS rating with the overall client experience of women, combining all domain responses. This 10-point VAS rating was based on the recent recommendations of the National Patient Survey Coordination Centre [33]. The overall client experiences score was obtained by first computing an average score per domain (where the 1, 2,3 or 4 response was treated numerically), and then computing an unweighted average across the 8 domain scores, resulting in an overall experience score with range 1–4. The association of women's global rating with their experience as a client was expressed by Spearman's correlation coefficient (rho).

The last step was a preliminary assessment of the discriminative validity of the ReproQ by three so-called known group comparisons. The client experience was compared applying the following groupings: 1) pregnant women versus women who recently had given birth; 2) women with better vs. worse clinical outcome of their baby depending on perceived health problems by the mother and hospitalization of the baby (altogether 4 groups); and 3) women who received care in fully integrated facilities versus women who received care in less integrated facilities.

We calculated domain scores (giving a profile) and an overall ReproQ score. Domain scores were declared missing when less than half of the items of that domain were filled out. We refrained from imputation of missing data. If more than half of the domain scores were missing, no overall score was computed. Because the experience data did not show a normal distribution, we report the overall median (MD) and the interquartile range (IQR) of all Responsiveness domains. To explore if differences in performance were significant between groups, we performed a Mann-Whitney test or Kruskal-Wallis test depending on the number of determinant categories (2 or 4, respectively). Significance level was p<0.05, without adjustment for multiple testing, as this was an explorative study, without prior sample size calculation. For the statistical analyses we used SPSS 21.0.

### General

The development process was supervised by a steering committee. This group consisted of representatives from health professionals, health insurance companies, a client-patient association, and members of the research team. Besides the steering committee, we were advised by a senior officer of the WHO engaged in the development of responsiveness measurement, with sufficient knowledge of the Dutch language.

The Medical Ethical Review Board of the University Medical Centre Utrecht approved the study protocol (study number MEC-2012–435).

## Results

### Item generation

The test version of our antepartum questionnaire contained 30 experience items. The postpartum questionnaire contained 36 experience items. The difference is explained by items in the domains Prompt Attention and Basic Amenities concerning specific elements of the delivery and postnatal care, such as the facilities during hospitalization after the delivery or the presence of a maternity nurse.

### Interviewing stakeholders to determine the content validity

The mean age of the participating women was 32.3 years (SD = 5.5). Of the 21 women, 6 reported to be of non-Dutch origin (29%). Most women had a high education; 8 women had a low/middle education (38%). All women were married or living together. Half of the women gave birth for the first time (52%). 13 of the 21 women received care in an integrated facility (62%). The characteristics are described in Table 1. All responsiveness domains were confirmed as being relevant in general. The domains Dignity and Communication were selected as most important by clients, by health professionals from their own perspective, and from the proxy-perspective of clients with low educational level or migrant status as expressed by these professionals. Clients and health professionals gave altogether 266 comments about the items in the Responsiveness domains (roughly 1 out of 5 items received a comment). 93 (35%) of these comments were related to the clarity of the wording of items. The participants stated problems with specific terms e.g. “personal attention”, “home situation” and the meaning of “several options” in the item “Could you choose from several options for postnatal care?”.

**Table 1:** Characteristics of the participating women in the preparatory interview study and the ReproQ survey study.

Characteristics		Interview study	Survey study	
		% (N = 21)	Antepartum % (N = 351)	Postpartum % (N = 433)
Age	≤25	19	5	8
	26–30	29	32	29
	31–35	24	38	42
	> = 36	29	21	16
	Missing	0	4	5
Ethnic background	Dutch	71	85	79
	Non-Dutch	29	9	9
	Missing	0	6	12
Education	Low	0	1	1
	Middle	38	40	36
	High	62	54	57
	Education abroad	0	0	2
	Missing	0	5	4
Marital status	Married/living together	100	92	91
	Relationship, not living together	0	3	3
	No relationship	0	1	2
	Missing	0	5	4
Parity	Primiparous	48	51	49
	Multiparous	52	45	47
	Missing	0	3	4
Integrated care	Integrated care facility	71	46	52
	Non-integrated care facility	29	49	44
	Missing	0	4	4
Maternity care organization	1	52	23	25
	2	29	24	27
	3	-	39	23
	4			
	Missing	-	4	5

Of the 266 comments, 119 comments (45%) concerned the relevance of items. Women noted difficulty in giving response if they had not been in a situation as described. Health professionals doubted whether some items could be judged by clients in case of high urgency of the care provided. They suggested adaptations of question or response (adding “not applicable”) in some instances.

54 comments (20%) suggested literal improvements in text of items or the response.

The topics claimed more than once to be missing were the client's judgment of the health professional's expertise and specific items on cultural customs and traditions of migrant women. As the ReproQ is to be used in connection to medical outcome measures, we refrained from adding an item on assigned expertise.

The suitability for women with low educational level was judged as sufficient by 10 of the 18 health professionals, while one health professional thought the questionnaire was unsuitable for women with low education. All professionals emphasized to be cautious with the application of standard survey data collection techniques in respondents with a low educational level.

Based on all comments, we left 11 items unchanged; 7 items were slightly rephrased; 5 items were deleted; 2 items were added; and the response mode of 10 items was rephrased (adding “not applicable”).

### Survey study to determine psychometric characteristics

The characteristics of pregnant women and women who recently had given birth are presented in Table 1. As differences were minimal, characteristics are described combined. The participating women had a mean age of 33.1 years (SD = 4.4). Of the 784 women who responded, 72 (9%) reported to be of non-Dutch origin. 71 women were not living together with the father of the child, or did not have a relationship with the father at all (9%).

The response pattern of the women generally showed high responsiveness to the client. The response modus “never” (representing an adverse experience) was not used in several items. “Never” was most often used in the item concerning “choice of health care professional” (19.7%) in the antepartum and postpartum questionnaire (18.2%). The response modus “always” was least often used in the item concerning waiting in the antepartum questionnaire (20.3%), and most by the item concerning privacy (94.3%). In the postpartum questionnaire, the response modus “always” was least often used in the item concerning the furnishing of the maternity care organizations (36.1%), and most often in the item “treated with respect” (88.6%). The per item missing rates were all below 5%. Filling out the antepartum questionnaire lasted on average 16 minutes (95% confidence interval (CI): 11–21min). The postpartum questionnaire took on average 14 minutes (95% CI: 11–17min).

The EFA revealed 9 factors in the antepartum questionnaire; 7 factors in experience with delivery, and 5 factors in postnatal care. Table 2 shows the factor loadings of each item (after rotation) for pregnancy, labour and birth, and postnatal care phase separately. Factor loadings of items that deviate from the dominant factor (i.e. the domain on which most of the items of the domain loaded) are shown in italics. The factors that included two items or more had a Cronbach's alpha varying between 0.68 and 0.89. From the EFA it appears that the factor solution shows considerable commonality across the three phases.

**Table 2:** Exploratory factor analysis of the ReproQ (antenatal, delivery, and postpartum responses separately).

Domain	Item	Adap-tation	Factor number and factor loading*		
			Antepartum (N = 351)	Delivery (N = 433)	Postpartum (N = 433)
Dignity	Respecting privacy**	NC	F1–0.46	F1–0.52	F4–0.59
	Treating with respect	NC	F1–0.81	F1–0.74	F1–0.81
	Giving personal attention	NC	F1–0.67	F1–0.61	F1–0.66
	Treating with kindness	NC	F1–0.81	F1–0.72	F1–0.78
Autonomy	Involving client in decision-making	NC	F7–0.80	F3–0.70	F4–0.63
	Acceptance of treatment refusal	NC	F7–0.82	F3–0.70	F4–0.69
	Considering personal wishes regarding pregnancy and birth	DD	F1–0.64	*F1–0*.*48* F3–0.58	*F1–0*.*55* F4–0.48
	Offering Down's syndrome screening as free choice	NC	F9–0.86	-	-
	Involving client in decision-making on pain relief	NC	-	F7–0.80	F5–0.83
	Involving client in decision-making on setting of birth	AI AR	F3–0.35 F5–0.36	F6–0.67	F5–0.50
Confidentiality	Trustworthy as health professional	DD	F1–0.64	F1–0.59	F1–0.62
	Secured provision of medical information to others	DEL	F4–0.32	F4–0.42 F5–0.54	F3–0.45 F4–0.49
Communication	Responsive to client questions	NC	F1–0.46	*F1–0*.*43* F4–0.48	F1–0.53 *F2–0*.*53*
	Consistency of advice across professionals	NC	F1–0.46	F4–0.65	F1–0.47
	Comprehensibility of explanation	NC	F5–0.55	F4–0.77	F1–0.50
	Provision of information while treated	NC	F5–0.57	*F1–0*.*43* F4–0.48	F1–0.46 *F2–0*.*42*
Prompt attention	Access for appointment/contact in urgent situations	AR	F8–0.63	F2–0.65	*F1–0*.*45 F2–0*.*42* F3–0.46
	Access for appointment/contact without urgency	NC	*F2–0*.*37* F4–0.38 *F8–0*.*39*	F2–0.59	*F1–0*.*42 F2–0*.*40* F3–0.47
	Waiting time for service	AI AR	F4–0.57	F2–0.43	F3–0.62
	Physical accessibility of setting	AI	F4–0.65	F2–0.65	F3–0.57
	Prompt phone response of health professional	AI	F4–0.79	F2–0.71	F3–0.48
Social consideration	Attention for family	DEL	F2–0.80	F3–0.59	F2–0.65
	Taking into account of family duties when making appointments	AI	F2–0.76	F3–0.57	F2–0.71
	Involvement of the partner in care provision	NC	F2–0.76	F3–0.47	F2–0.70
Basic amenities	Decoration of setting	AI	F3–0.68	-	-
	Hygiene of setting	AI	F3–0.75	-	-
	Comfort of setting	AI	F3–0.66	-	-
	Playground children or other facilities	DEL	F3–0.46	-	-
Choice and continuity	Making service time available on request of the client	DD	-	F2–0.55	*F2–0*.*53* F3–0.50
	Continuity of care provision when change of individual professional (same discipline)	AI AR	F6–0.77	F5–0.53	F3–0.62
	Continuity of care provision when change of professional (across disciplines)	AI AR	F6–0.84	F5–0.60	F3–0.67
	Allowance for selecting a preferred type of health professional	NC	F5–0.66	F6–0.63	F3–0.43
	Being explicit on which health professional is actual in charge	NC	F6–0.61	F4–0.49	F2–0.60

Adaptation: NC = no change; AI = adjusted item; AR = adjusted response mode; DD = assigned to different domain based on EFA; DEL = removed

* The last three columns represent 3 separate factor analyses. The number of the factor is listed in the order of the output (F1 = first factor, F2 = second factor, etc). Only results with a factor loading > 0.3 are shown. If an item corresponds to a factor numbers in italics, then the factor analysis apparently placed the item into another domain, as we assumed constructing the item cf. the WHO domain structure.

** For reasons of brevity, we indicate the contents of each item as a professional or maternity care organization characteristic. The question to the respondent refers to the actual experience. E.g. the first item indicated with “respecting privacy”, in the ReproQ reads as “Did the caregiver consider your privacy during care provision?”

**Figure 2:**
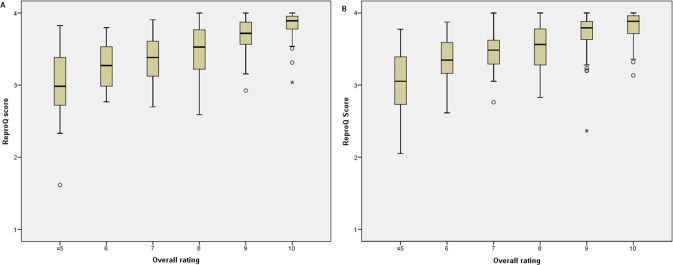
Convergent validity: association between overall rating of maternity care, and ReproQ score (all domains combined). Fig. 2A shows the results during the antenatal phase; Fig. 2B shows the results during labour and postnatal care. The overall rating (10-point VAS scale) was significantly associated with the overall ReproQ score (i.e. the unweighted summation [range 1–4] of the individual eight domains), in both the antepartum (p<0.001) and the postpartum phase (p<0.001).

The median score was 3.69 for the antepartum version (IQR 3.39–3.87). The median score of the postpartum version was 3.74 (IQR 3.45–3.88). In Fig. 2 the global 10-point VAS rating was related to the overall ReproQ client experience score, to determine the convergent validity. A low VAS rating was associated with a lower ReproQ score in both the antepartum (r = 0.59; p<0.001) and the postpartum questionnaire (r = 0.56; p<0.001).

**Figure 3:**
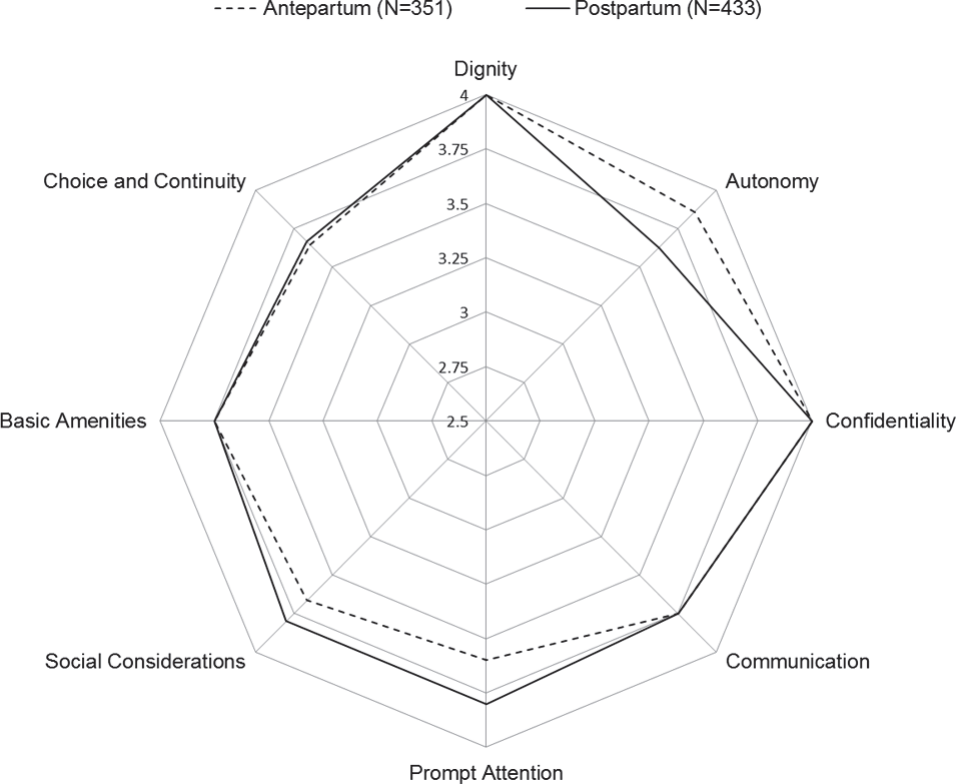
Discriminative validity: median domain-specific ReproQ-score, for antepartum and postpartum questionnaire. The figure shows the median domain-specific ReproQ score for antepartum (A) and postpartum (P) questionnaire. The interquartile range of the domains are as follows: Dignity: 3.8–4.0 (A); 3.7–4.0 (P); Autonomy: 3.4–4.0 (A); 3.3–4.0 (P); Confidentiality 4.0–4.0 (A); 3.5–4.0 (P); Communication: 3.5–4.0 (A); 3.5–4.0 (P); Prompt Attention: 3.4–3.8 (A); 3.4–4.0 (P); Social Consideration: 3.0–4.0 (A); 3.4–4.0 (P); Basic Amenities 3.3–4.0 (A); 3.3–4.0 (P); Choice and Continuity: 2.9–4.0 (A); 3.1–4.0 (P). The average score combining all domains per individual has a median of 3.68 (IQR = 3.40–3.87) antepartum, and a median of 3.73 (IQR = 3.44–3.88) postpartum (p = 0.23). Domain-wise, Autonomy, Respect, Confidentiality were experienced better in pregnant women compared women who recently had given birth (p between 0.021 and <0.0001). Women who recently had given birth had better experiences with Prompt Attention, Social Consideration and Choice and Continuity (p between 0.033 and <0.0001).

The results on discriminative validity showed that overall, pregnant women and women who recently had given birth had a similar overall ReproQ score (MD = 3.68, IQR = 3.40–3.87 vs. MD = 3.73, IQR = 3.44–3.88; p = 0.23), see Fig. 3. Domain-wise, Autonomy in pregnant women was experienced better compared to Autonomy in women who recently had given birth. Women who recently had given birth had better experiences with Prompt Attention.

**Figure 4:**
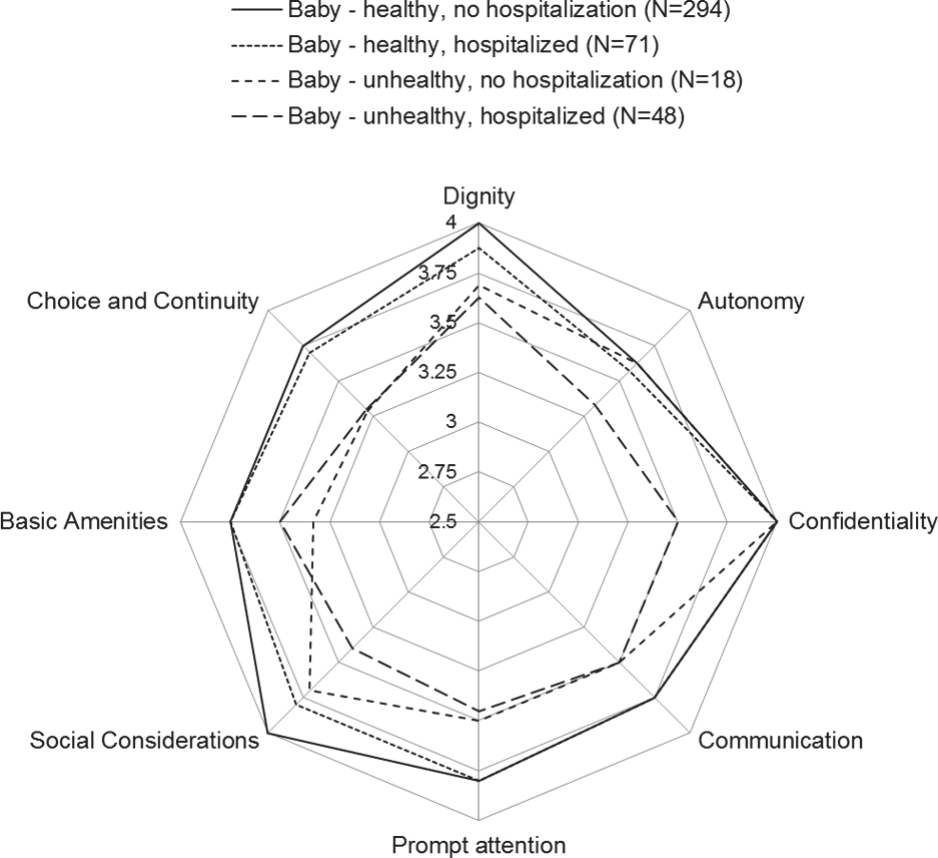
Discriminative validity: median domain-specific ReproQ-score according to perceived neonatal outcome and neonatal hospital admission. The figure shows the median domain-specific ReproQ score for 4 groups: 1) women who did not perceive health problems with their baby and whose baby was not hospital-admitted (MD = 3.78, IQR = 3.52–3.87); 2) women who did not perceive health problems with their baby, but whose baby was hospital-admitted [e.g. for monitoring] (MD = 3.72, IQR = 3.45–3.85); 3) women who perceived health problems with their baby, but the baby was not hospital-admitted (MD = 3.47, IQR = 3.16–3.80); and 4) women who perceived health problems with their baby and whose baby was hospitalized (MD = 3.42, IQR = 3.10–3,72) (p>0.001). Note that the client's perception of neonatal outcome may differ from clinical judgement; here we assume the client's perspective to be primarily important.

Women who perceived no health problems in their baby reported best overall ReproQ score (see Fig. 4), independent whether their baby was hospitalized (MD = 3.78, IQR = 3.52–3.87) or not (M = 3.72, IQR = 3.45–3.85). Within these groups, women with their baby hospitalized showed more negative experiences concerning Dignity and Social Considerations than women whose baby was not hospitalized. Women who did perceive health problems in their baby, but whose baby was not hospitalized showed a lower median overall score (MD = 3.47, IQR = 3.16–3.80). Women whose baby had been hospitalized with (perceived) health problems showed the lowest overall scores (MD = 3.42, IQR = 3.10–3.72). All domains and the overall score differed significantly between the four subgroups (Kruskal-Wallis, all P<0.001).

**Figure 5:**
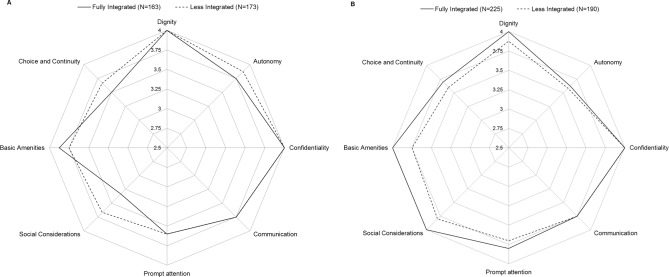
Discriminative validity: median domain-specific ReproQ-score according to integration level of care facilities. The figures show the median domain-specific ReproQ score during the antenatal phase (5A) en during labour and postpartum care (5B), for women who received care in fully integrated organizations and for women who received care in less integrated organizations. These differences were not significant during antenatal care (p = 0.142), but were significant in care during labour and birth, and during postnatal care (p<0.001).

During pregnancy, the overall ReproQ score of women who received care in a full integrated facility (MD = 3.65, IQR = 3.37–3.86) showed no significant difference with women who received care in a less integrated facility (MD = 3.74, IQR = 3.42–3.88; p = 0.14) (see Fig. 5). In the delivery and postpartum phase women who received care in integrated facilities had a slightly higher score compared to less integrated facilities (Md = 3.78, IQR = 3.53–3.90 vs. Md = 3.63, IQR = 3.34–3.84; p<0.001). All domains except “Choice and continuity” (p = 0.062) differed significantly.

Based on the results of the statistical analyses we left 19 items unchanged; 10 items were slightly rephrased; 3 items were deleted; 4 new items from the original item pool were added; 3 items were left unchanged but formally assigned to another domain; and the response mode of 4 items was slightly rephrased (see Table 2). No changes were needed after the second round of stakeholders experts for final verification that wording of item and response were strictly neutral and unequivocal for types of organizations. The questionnaires can be accessed online: antepartum version, postpartum version.

## Discussion

We developed a self-report questionnaire, the ReproQ, to measure the performance of maternity care from the perspective of clients. We used the WHO responsiveness model, which evaluates experienced client interactions with professionals and the care providing organisation. Content validity of the instrument was judged appropriate with balanced contribution of the WHO domains. According to participants, more attention could be given to sensitivity for the cultural background and traditions of the client, and the experienced professional expertise. The null version of the questionnaire was adjusted based on all comments, but we did not include a domain on the experienced expertise as in our opinion this should be primarily reflected in the clinical outcome, which is difficult to assess by the client.

The construct validity of the improved ReproQ version 1 was established in a survey study involving pregnant and recently delivering women. The response pattern showed overall good to excellent overall ReproQ scores, averaging over domains. The effect that participants rarely use the most negative response modus, is known from other self-report instruments in maternity care and may be partially caused by the fact that pregnancy and childbirth are not a disease and generally have good outcome [23,34–36]. The most positive response category (“always”) was used the most, demonstrating a ceiling effect also shown in the maternity experience survey of the National Perinatal Epidemiology Unit [23].

The exploratory factor analyses largely confirmed the pre-stated domain structure. However, the EFA strongly suggested to rearrange and reword items from the Confidentiality domain, because these items loaded on different factors for the different stages of maternity care. Testing convergent validity, we established a clear association between the overall VAS rating and the overall ReproQ score.

The known group comparisons revealed literature-expected differences between women perceiving good vs. bad outcomes in their baby, being aware that this may be a “cross-over” effect rather than actually reflect poor responsiveness. In clients who received care in fully integrated facilities vs. less integrated facilities, we observed differences during birth and postnatal care, but not during pregnancy as might be expected as integration effects from the perspective of the client is most clearly experienced at that stage.

### Strengths

The ReproQ focuses on the actual experiences of women with maternity care while existing questionnaires mainly focus on procedural aspects [22–24]. While following the adequate procedure can contribute to responsiveness, it does not replace or predict care provision which is client-centered. For example, the provision of written information can be a valuable standard procedure, but it requires verification of utilization and understanding of the information.

The ReproQ is unique in the coverage of the eight responsiveness domains, which were all considered valuable. The questionnaire of the National Health Service in the United Kingdom included only 6 of the 8 Responsiveness domains, often using one specific item within a domain. Prompt Attention was e.g. indicated by the item “were you given the help you needed?”[22]. This item was similar in the questionnaire of the National Perinatal Epidemiology Unit concerning women's experience with maternity care [23]. As it combines promptness and perceived adequacy, response is difficult to interpret.

To prevent cross-over effects from labour and birth to the antenatal experiences [28], we created two separate questionnaires to measure the experiences during pregnancy and the experiences during delivery and postpartum care. This facilitates quality improvement as the services involved usually are different.

### Limitations

This study had several limitations.

First, fewer clients participated in the group interviews than anticipated. In order to cover all relevant perspectives and to maximize input on the issue of comprehensibility for the deprived, we conducted additional individual interviews. In both forms all participants first wrote down their individual comments (positive/negative/change) on contents and readability for each questionnaire item of the ReproQ core separately. No discussion or exchange was allowed in the group session at this stage. In the group sessions, these items were then presented one by one, and discussed if asked for. The items were primarily adapted based on the detailed individual written comments, which frequently converged; occasionally the plenary discussion was used to solve an arbitrary wording choice. We assume the combination of group and individual sessions did not compromise the results.

Second, there is no reference standard available to measure performance from the perspective of clients, which makes it hard to establish the quality of the measured concept. We believe however that the responsiveness model provides a solid conceptual base, confirmed by extensive testing during its development and thereafter [6,13]. The comprehensiveness and cross-cultural suitability has been confirmed in our study.

Third, women with a low educational level were underrepresented in our studies despite repeated and considerable efforts to engage them. An explanation may be lack of perceived control of these women, which is reflected in reluctance to participate: they do not believe that participation or responding matters [37].

Fourth, a minority of the non-western women participated. This percentage (9%) is lower than the percentage of non-Dutch pregnant women in the Netherlands (non-Dutch: 16%) [38]. Possible explanations include a language barrier [39,40] and our reliance on an anonymous digital procedure. Perhaps the frequent coexistence of low education and non-Western ethnicity plays a role [41]. To increase their participation, the questionnaire could be adapted by adding specific questions or by translating the questionnaire into other languages. For both non-response prone groups the questionnaire could also be presented differently. For example, with assistance of an independent third person, or by using a face-to-face interview. Another option would be to ask key figures of their local society to promote participation.

### Future use

The resulting questionnaire may be used in various types of evaluation studies, dedicated to compare specific interventions or specific organization structures, or health care providers.

From its conceptual base–a complement to medical outcome–it follows that outcomes, like mortality of both mother and child, or compound measures like the Perinatal Adverse Outcome Index [42] are unconditionally required for overall judgement. Interpretation of the relevance of average ReproQ differences requires further study.

### Future research

Further research Is needed on the discriminative capacity of the ReproQ to show differences between care providers, and on the interpretation and relevance of observed differences. Also, testing the proposed domain structure in a new sample using confirmatory factor analysis is needed.

## Conclusion

We developed a client experience questionnaire (“ReproQ”) to measure maternity care performance based on the WHO responsiveness model. After content analysis the improved ReproQ questionnaire showed acceptable convergent and satisfactory discriminative validity. Participation of disadvantaged groups in measurement of client experiences may require additional approaches.

## Supporting Information

S1 Table The number of participants for each organization in the interviews. (DOCX)
