# Supplementary material for: Measuring Client Experiences in Maternity Care under Change: Development of a Questionnaire Based on the WHO Responsiveness Model
Source: PLoS One. 2015 Feb 11;10(2):e0117031. doi: 10.1371/journal.pone.0117031 (PMC4324965; doi:10.1371/journal.pone.0117031)
Supplement: S1 Table — (DOCX) [file pone.0117031.s001.docx]

**Table S1: The number of participants for each organization in the interviews**

|  | Maternity care organization 1* | Maternity care organization 2* | Maternity care organization 3* |
| --- | --- | --- | --- |
| Group interview - antepartum | 5 | 4 | 0 |
| Group interview - postpartum | 2 | 2 | 3 |
| Individual interview - ante/postpartum | 4 | 0 | 1 |
| Experts - ante/postpartum | 8 | 7 | 6 |

* Description of the participating maternity care organizations:

1) an integrated midwifery practice and clinic from the university hospital (Utrecht)

2) a clinic from the university hospital, and a postnatal birth centre (Rotterdam)
3) an integrated midwifery practice and clinic from a peripheral hospital (Roosendaal)
